# Multi-pathogen epidemiological survey of subclinical mastitis, gastrointestinal parasitism, and zoonotic diseases in smallholder dairy cattle in South Sulawesi, Indonesia

**DOI:** 10.14202/vetworld.2026.2619-2633

**Published:** 2026-06-28

**Authors:** Fika Yuliza Purba, Anak Agung Putu Joni Wahyuda, Muhammad Ardiansyah Nurdin, Muhammad Muflih Nur, Anjani Marisa Kartikasari, Zulfikri Mustakdir, Irwan Ismail, Erick Vitus Gabriel Komba, Emely J. Escala, Subaedy Yusuf

**Affiliations:** 1 Veterinary Medicine Study Program, Faculty of Medicine, Universitas Hasanuddin, Makassar 90245, Indonesia; 2 Animal Production and Health Research Group, Universitas Hasanuddin, Makassar 90245, Indonesia; 3 Center for Research and Development of Livestock Resources and Tropical Animals, Universitas Hasanuddin, Makassar 90245, Indonesia; 4 Department of Veterinary Medicine and Public Health, Sokoine University of Agriculture, P.O BOX 3021, Chuo Kikuu, Morogoro, Tanzania; 5 Livestock Research and Development Center, Capiz State University, Capiz 5802, Philippines; 6 Agricultural Science Doctoral Program, Postgraduate School, Universitas Hasanuddin, Makassar 90245, Indonesia

**Keywords:** bovine tuberculosis, brucellosis, dairy cattle, gastrointestinal parasites, Indonesia, smallholder farming, subclinical mastitis, zoonotic diseases

## Abstract

**Background and Aim:**

Smallholder dairy cattle systems in Indonesia face persistent health challenges that compromise productivity, milk quality, and public health. This study aimed to investigate the prevalence of subclinical mastitis, gastrointestinal parasitism, and selected zoonotic diseases (*Mycobacterium bovis* and *Brucella abortus*) in smallholder dairy cattle in Enrekang Regency, South Sulawesi, a key dairy production center in eastern Indonesia.

**Materials and Methods:**

A cross-sectional survey was conducted in July 2025 across 29 smallholder farms involving 94 dairy cattle. Serological screening (enzyme-linked immunosorbent assay for bovine tuberculosis (bTB) and Rose Bengal Test for brucellosis) was performed on 90 animals. Subclinical mastitis was assessed in 73 lactating cows (289 quarters) using the California Mastitis Test (CMT), with CMT-positive samples (score ≥2) subjected to bacteriological culture and matrix-assisted laser desorption/ionization time-of-flight mass spectrometry identification. Gastrointestinal parasites were examined in 94 fecal samples using direct smear, flotation, and sedimentation techniques. Descriptive statistics and 95% confidence intervals (CIs) were calculated.

**Results:**

All serum samples tested negative for bTB and brucellosis. Subclinical mastitis prevalence was high: 38.75% at quarter level (112/289; 95% CI: 33.2–44.6%), 75.34% at cow level (55/73; 95% CI: 64.2–84.1%), and 86.21% at herd level (25/29; 95% CI: 68.8–95.0%). Dominant bacterial isolates included *Streptococcus uberis* (16.7%), *Streptococcus agalactiae* (13.3%), and *Staphylococcus aureus* (13.3%). Gastrointestinal parasitism affected 75.53% of cattle, with strongyle-type eggs (39.36%), *Strongyloides* spp. (30.85%), *Eimeria* spp. (21.28%), and *Fasciola* spp. (14.89%) most prevalent. Co-infection of mastitis and parasitism occurred in 56.16% of lactating cows.

**Conclusion:**

This first multi-pathogen survey in Enrekang Regency revealed the absence of major zoonotic bacterial diseases alongside a high burden of subclinical mastitis and gastrointestinal parasitism. These findings highlight the predominance of endemic, productivity-limiting conditions in smallholder dairy systems and underscore the need for integrated control programs focusing on milking hygiene, housing sanitation, and strategic parasite management.

## INTRODUCTION

Dairy production is a central component of smallholder livelihoods across Indonesia, contributing significantly to rural income generation, household nutrition, and food security [1]. In regions such as South Sulawesi, smallholder dairy systems play an increasingly important role in meeting local milk demand. However, these systems remain vulnerable to a range of infectious and production-limiting diseases that adversely affect animal health, farm profitability, and product quality. Such health challenges compromise animal welfare and productivity while simultaneously posing risks to public health through zoonotic transmission and the production of lower-quality milk. Addressing these issues is therefore essential for improving livestock productivity, strengthening food safety, and supporting the long-term sustainability of rural dairy enterprises [2, 3].

Among the most important health constraints in dairy cattle is mastitis, particularly the subclinical form, which often remains undetected despite causing substantial economic losses. Subclinical mastitis reduces milk yield by approximately 10%–30% per affected cow and negatively affects milk quality, resulting in significant financial consequences for dairy producers [4]. Several factors contribute to the high prevalence of mastitis in smallholder dairy systems, including poor milking hygiene, inadequate udder sanitation, and limited implementation of good dairy farming practices [5, 6]. Despite extensive investigations conducted in Java and several other Indonesian provinces, epidemiological information regarding mastitis in eastern Indonesia remains limited. Equally important are bovine tuberculosis (bTB) and brucellosis, two zoonotic bacterial diseases that have major implications for both animal and human health [7]. bTB reduces productivity and poses occupational health risks to farmers and animal handlers, whereas brucellosis, caused by *Brucella abortus*, is associated with reproductive disorders in cattle and febrile illness in humans.

Regional estimates indicate that brucellosis prevalence in South Sulawesi may reach approximately 11% in certain cattle populations, although reported prevalence varies with diagnostic methods, study designs, and geographic locations [8, 9]. These variations emphasize the need for localized surveillance and highlight the potential for under-detection in field-based investigations. In addition to bacterial diseases, endoparasitic infections remain a persistent challenge in tropical dairy production systems. Gastrointestinal parasites such as strongyles, *Strongyloides* spp., *Eimeria* spp., and *Fasciola* spp. thrive under humid environmental conditions and traditional management practices, leading to reduced feed efficiency, impaired growth, decreased milk production, and overall productivity losses. Previous studies have reported strongyle-type infections in South Sulawesi with a prevalence approaching 49%, although prevalence may vary substantially depending on environmental and management factors [10]. Furthermore, the concurrent occurrence of parasitic and bacterial infections may exacerbate production losses and compromise animal health in smallholder dairy systems.

Enrekang Regency, located in South Sulawesi Province, represents one of the emerging dairy production centers outside Java and is characterized by smallholder-based dairy management systems and increasing demand for milk. These characteristics make the region an important epidemiological setting for understanding disease dynamics in developing dairy production areas. However, official statistics indicate a considerable decline in the dairy cattle population, from 1,011 heads in 2020 to 612 in 2024, accompanied by a corresponding reduction in milk production [11]. Based on provisional records, the dairy cattle population in 2025 was estimated at 651 heads [12]. This decline may reflect the cumulative effects of disease burden, suboptimal management practices, and economic constraints, with chronic conditions such as mastitis and gastrointestinal parasitism potentially contributing to reduced productivity and herd performance.

A substantial knowledge gap remains regarding the epidemiological status of multiple infectious and parasitic diseases affecting dairy cattle in eastern Indonesia. Most available studies have focused on individual diseases or have been conducted in major dairy-producing regions of western Indonesia, particularly Java. Consequently, comprehensive information describing the simultaneous occurrence of zoonotic bacterial diseases, mastitis, and gastrointestinal parasitism in smallholder dairy systems of South Sulawesi is lacking. Furthermore, few studies have integrated serological, microbiological, and parasitological diagnostic approaches within a single epidemiological survey. The absence of such integrated investigations limits the understanding of the overall disease burden affecting dairy cattle and restricts the development of evidence-based, region-specific disease control strategies. This knowledge gap is particularly important in emerging dairy-producing areas such as Enrekang Regency, where disease-associated productivity losses can have substantial economic consequences for smallholder farmers and influence public health through potential zoonotic transmission.

Therefore, this study aimed to investigate the prevalence of selected infectious and parasitic diseases in smallholder dairy cattle in Enrekang Regency, South Sulawesi. Specifically, the study sought to determine the serological status of bTB and brucellosis, estimate the prevalence of subclinical mastitis at quarter, animal, and herd levels, identify bacterial species associated with mastitic milk using matrix-assisted laser desorption/ionization time-of-flight mass spectrometry (MALDI-TOF MS), and characterize the prevalence of gastrointestinal endoparasitic infections. These conditions were selected because of their relevance to animal health, public health, and dairy productivity, as well as their suitability for field-based epidemiological assessment. Other important conditions, including reproductive disorders such as repeat breeding, were beyond the scope of the present investigation and warrant future study. Accordingly, this study was designed as an exploratory baseline epidemiological survey to generate descriptive data on the prevalence and etiology of selected infectious and parasitic diseases in smallholder dairy cattle in South Sulawesi rather than to identify or model specific risk factors.

## MATERIALS AND METHODS

### Ethical approval

Ethical approval for this study was obtained from the Ethics Committee, Faculty of Medicine, Universitas Hasanuddin, Indonesia (Approval No. 009/UN4.1.RSHUH/B/PP36/2025). All study procedures were conducted in accordance with institutional animal care and welfare guidelines.

Participation of farmers was entirely voluntary. Before sample collection, the objectives, procedures, potential benefits, and minimal risks associated with the study were explained to farm owners, and written informed consent was obtained from all participants.

All animal-handling procedures adhered to accepted animal welfare principles. Jugular venipuncture and rectal fecal collection were performed by trained personnel using minimal restraint techniques. Animals were manually handled by experienced assistants to minimize stress and discomfort. No sedation or analgesia was required because all procedures were brief, minimally invasive, and considered low-stress. No invasive interventions beyond routine blood and fecal sample collection were performed.

Standard biosafety measures, including the use of personal protective equipment, were implemented during both field sampling and laboratory procedures to reduce the risk of zoonotic exposure for personnel. Upon completion of the study, participating farmers were provided with feedback regarding herd health findings and practical recommendations for disease prevention, biosecurity improvement, and herd health management.

### Study period and location

**Figure 1:**
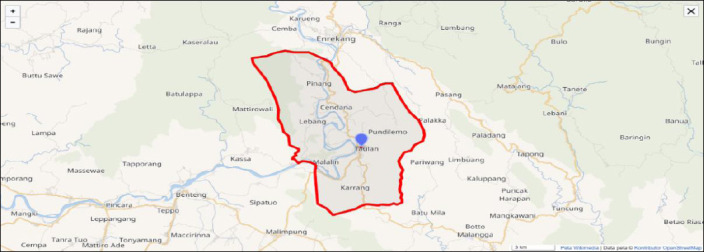
Map of the study area in Enrekang Regency, South Sulawesi, Indonesia. The red boundary indicates the study area (Cendana Sub-district). Pinang and Lebang villages were conveniently selected for the study. The map was adopted from Wikipedia using the Kartographer mapframe/OpenStreetMap data. [Source: https://id.wikipedia.org/wiki/Cendana,_ Enrekang. Accessed on 14 June 2026].

This study was conducted from July 1 to July 31, 2025, in Cendana Sub-district, Enrekang Regency, South Sulawesi Province, Indonesia, located at 3°39′20.15197″S and 119°47′16.76782″E (Figure 1). The study period corresponded to the dry season in South Sulawesi. The average annual temperature in the region ranges from 22°C to 30°C, providing favorable environmental conditions for dairy production and the persistence of infectious and parasitic agents.

Enrekang Regency is recognized as one of the major dairy-producing areas in South Sulawesi, where smallholder dairy farming serves as an important source of household income and livelihood. The dairy production system is predominantly based on smallholder farms, typically maintaining fewer than 10 lactating cows per household under traditional housing and hand milking practices. Cattle were generally confined and managed using a cut-and-carry feeding system. Common forage sources included Napier grass (*Pennisetum purpureum*) and other locally available grasses. Water for livestock was primarily obtained from groundwater sources and nearby rivers.

### Study design and study population

According to the National Livestock Census [11], Enrekang Regency has a dairy cattle population of 612 animals, with the highest concentration located in Cendana Sub-district. Administratively, Cendana Sub-district comprises seven villages: Cendana, Karrang, Lebang, Malalin, Pinang, Pundilemo, and Taulan. Based on a previous census-based enumeration conducted in the same area [13], dairy cattle were distributed across five villages, namely Pinang (19 farms), Cendana (15 farms), Lebang (10 farms), Karrang (2 farms), and Pundilemo (2 farms), with a total of 48 smallholder dairy farms and an actively managed cattle population of approximately 267 animals. This localized estimate was considered more representative of the accessible study population and was therefore used as the practical sampling frame.

A cross-sectional study was conducted at both farm and animal levels. Owing to logistical considerations and the clustering of dairy production, farms were selected using a non-probability convenience sampling approach from the two villages with the highest concentration of dairy farms, namely Pinang and Lebang. A total of 29 farms were enrolled in the study. Inclusion criteria were the presence of at least one lactating cow, no history of antimicrobial treatment in the preceding 14 days, and the farmer’s willingness to participate, as evidenced by informed consent.

This investigation represents original fieldwork conducted in Cendana Sub-district using a multi-diagnostic approach comprising ELISA and Rose Bengal Test (RBT) for zoonotic disease surveillance, quarter-level California mastitis test (CMT) screening for subclinical mastitis, bacterial identification using MALDI-TOF MS, and multi-method fecal parasitological examination. The study incorporated multiple epidemiological levels of assessment, including quarter-level evaluation for mastitis, individual animal-level evaluation for serological and parasitological examinations, and herd-level assessment of management and housing characteristics. To the best of our knowledge, such an integrated multi-pathogen epidemiological survey has not previously been conducted in this setting.

Sample size was calculated using a single-proportion formula with finite population correction at a 95% confidence level. For subclinical mastitis, assuming an expected prevalence of 60% [6] and a desired precision of 10%, a minimum sample size of 60 lactating cows was required; however, 73 lactating cows were ultimately included. For zoonotic disease surveillance, assuming a prevalence of 10% [8, 14] and a precision of 5%, the minimum required sample size was 92 animals, whereas 90 animals were sampled. For gastrointestinal parasitism, assuming a prevalence of 46% [10] and a precision of 10%, a minimum sample size of 96 animals was required, and 94 animals were sampled. Based on the observed data, the prevalence of subclinical mastitis corresponded to a 95% CI of 64.4%–83.8% using the Wilson method. No zoonotic disease cases were detected (0/90), yielding a 95% CI of 0.0%–4.0% using the Clopper–Pearson exact method, indicating that low-level prevalence could not be completely excluded.

Finite population correction was incorporated because of the relatively small source population. However, clustering at the animal level was not considered in the sample size calculation. Consequently, no adjustment was made for intra-cluster correlation or design effect, which may have resulted in an underestimation of variance.

All lactating cows available during farm visits were included for mastitis screening, provided they had not received antimicrobial treatment within the preceding 2 weeks. Non-lactating cows and heifers were included for serological and fecal examinations when available and accessible, subject to farmer consent. These considerations were intended to ensure that the sampled population reasonably reflected the structure and management diversity of smallholder dairy systems in the study area.

A total of 90 cattle were tested serologically for bTB and brucellosis. Among these animals, 73 lactating cows provided quarter milk samples (n = 289) for CMT screening. Fecal samples were collected from 94 cattle for gastrointestinal parasite examination. Sampling included both adult cattle and calves because young animals, although excluded from serological and mastitis assessments, are epidemiologically important in gastrointestinal parasite transmission. Animal-level information, including age, parity, and lactation status, together with herd-level characteristics such as housing, flooring material, water source, manure management, forage and feeding practices, drainage conditions, and milking methods, was recorded to support descriptive epidemiological interpretation.

### Sample collection and diagnostic procedures

**Blood sampling and serum preparation**: Approximately 10 mL of jugular venous blood was collected aseptically from each animal using sterile vacutainer tubes without anticoagulant. Samples were transported under refrigerated conditions and delivered to the laboratory within 6 h of collection. Upon arrival, blood samples were allowed to clot at room temperature (22°C–24°C) and were subsequently centrifuged at 1267 × *g* for 15 min. Serum was harvested into cryovials and stored at −20°C until further analysis.

**Serological detection of *Mycobacterium bovis***: All serum samples (n = 90) were tested for antibodies against *M. bovis* using a commercial ELISA kit (Ring Biotechnology Co., Ltd., Beijing, China) as part of herd-level surveillance. Assays were performed according to the manufacturer’s instructions. Absorbance values were measured at 450 nm using a microplate reader. Each assay included manufacturer-provided positive and negative controls. Test results were considered valid only when control values fell within the recommended acceptance range.

Sample classification was based on the manufacturer’s recommended cutoff criteria using the sample-to-positive ratio, with values ≥0.30 considered positive for antibodies against *M. bovis*. Samples with values close to the cutoff threshold were conservatively classified as negative for prevalence estimation purposes.

According to the manufacturer, the assay exhibits analytical sensitivity exceeding 99% and specificity exceeding 95%. However, field-based diagnostic sensitivity for antibody detection of *M. bovis* may be substantially lower (approximately 60%–70%), particularly during early or latent stages of infection, whereas specificity generally remains above 97%. Therefore, negative results should be interpreted cautiously in the context of herd-level surveillance.

**Brucellosis**: All 90 serum samples were screened for antibodies against *B. abortus* using RBT. This assay was applied as a herd-level screening method and was not considered a definitive diagnostic test. Testing was performed in accordance with the World Organization for Animal Health (WOAH) Manual of Diagnostic Tests and Vaccines for Terrestrial Animals, following the protocol described by Morgan *et al*. [15, 16].

Approximately 30 μL of serum was mixed with an equal volume of Rose Bengal antigen (Veterinary Laboratories Agency, Weybridge, United Kingdom) on a glass plate and gently agitated for 4 min. Agglutination reactions were evaluated visually by trained laboratory personnel blinded to animal identity. Any visible agglutination was interpreted as a positive screening result.

The RBT is recognized as a highly sensitive screening assay, typically demonstrating approximately 95%–99% sensitivity, although specificity may vary due to cross-reactivity with other Gram-negative bacteria. Samples testing positive by RBT were predefined for confirmatory testing using the Complement Fixation Test, in accordance with WOAH recommendations. However, because all samples yielded negative RBT results, confirmatory testing was not required.

**Milk sampling and mastitis detection:** Milk samples were collected from all lactating cows included in the study. Before sampling, teats were cleaned with sterile cotton soaked in 70% ethanol to minimize contamination. Disposable towels were used individually for each cow, and teats were allowed to dry naturally after disinfection. Sampling was performed using a consistent teat order to reduce the risk of cross-contamination, and gloves were worn throughout the procedure. Approximately 10 mL of foremilk from each quarter was collected into sterile tubes after discarding the first few streams.

All quarter milk samples were initially screened for subclinical mastitis using CMT. Test reactions were scored semi-quantitatively as 0 (negative), trace, 1 (weak positive), 2 (distinct positive), or 3 (strong positive) based on the degree of gel formation after reagent mixing. Scoring was performed independently by two trained veterinarians, and any discrepancies were resolved through joint re-evaluation to ensure consistency.

In accordance with established diagnostic practices, a quarter was classified as CMT-positive when the score was ≥1, corresponding to somatic cell counts of approximately 200,000 to 500,000 cells/mL and indicating an increased likelihood of intramammary infection in dairy cattle [17, 18]. Quarters with CMT scores ≥2 were prioritized for bacteriological culture because these scores are more strongly associated with active infection and elevated somatic cell counts.

The CMT is widely used as a field-based screening tool for subclinical mastitis, with reported sensitivity ranging from approximately 75% to 95% and specificity ranging from 70% to 90% for detecting elevated somatic cell count thresholds (>200,000–400,000 cells/mL), depending on operator interpretation and selected cutoff criteria.

### Mastitis variables

Animal-level prevalence was defined as the proportion of cows with at least one CMT-positive quarter, whereas herd-level prevalence was defined as the proportion of farms with at least one affected cow. A CMT score ≥1 was used to estimate prevalence, whereas samples with CMT scores ≥2 were selected for bacteriological culture to improve pathogen recovery.

### Bacteriological culture and identification

Quarter milk samples classified as CMT-distinct positive (CMT score ≥2) were transported to the microbiology laboratory in insulated containers with ice packs and processed within 24 h. Approximately 10 μL of each sample was streaked onto blood agar and MacConkey agar plates and incubated aerobically at 37°C for 24–48 h. Blood agar consisted of tryptic soy agar supplemented with 5% defibrinated sheep blood. Plates showing no visible bacterial growth after 48 h of incubation were classified as “no growth” and were not re-cultured.

Colonies were sub-cultured to obtain pure isolates, and bacterial identification was subsequently performed using MALDI-TOF MS with the VITEK® MS system (bioMérieux, Marcy-l’Étoile, France). The application of MALDI-TOF MS for bacterial identification provided enhanced taxonomic resolution compared with conventional culture and biochemical approaches commonly used in previous Indonesian mastitis investigations.

For analysis, isolated colonies were prepared using the direct smear method and overlaid with α-cyano-4-hydroxycinnamic acid matrix. The preparations were air-dried before insertion into the instrument for spectral acquisition. Instrument calibration was performed using the manufacturer-provided reference standard in accordance with routine operating procedures.

Identification results were interpreted based on the confidence values generated by the VITEK® MS system [19]. Identifications with confidence values ≥99% were considered reliable at the species or genus level. Results with lower confidence values or classified as low discrimination were considered unreliable and were excluded from definitive identification. Standard aseptic procedures and routine laboratory quality control measures were applied throughout sample processing to minimize contamination.

### Fecal examination

Parasitological assessment in this study was limited to the qualitative detection of parasite eggs and oocysts; quantitative fecal egg counts were not performed. Consequently, infection intensity could not be determined.

Fecal samples were examined for gastrointestinal endoparasites using three standard parasitological techniques: direct smear, flotation, and sedimentation. Approximately 5–10 g of feces was collected directly from the rectum, stored at 4°C, and processed within 24 h of collection.

Flotation was performed using a saturated sugar solution with a specific gravity of approximately 1.27 at a feces-to-solution ratio of 1:10, followed by centrifugation at 880 × *g* for 10 min. Sedimentation was performed by centrifugation at 317 × *g* for 5 min. Parasites were identified primarily at the genus level based on egg and cyst morphology using standard parasitological identification keys [20]. Microscopic examination was conducted under a light microscope at magnifications of 100× and 400×.

Because quantitative fecal egg counts were not performed, the infection intensity could not be estimated, which should be considered a limitation of the study.

### Quality control in diagnostics

All diagnostic procedures were conducted under strict laboratory quality control standards in accordance with manufacturer instructions and WOAH guidelines. MALDI-TOF MS identifications were cross-validated using reference strains, and the instrument was calibrated daily before analysis to ensure analytical accuracy.

To assess repeatability, approximately 10% of samples were randomly selected for duplicate testing across diagnostic procedures. Agreement exceeded 95%. In cases of discrepant results, repeat testing was performed, and final outcomes were determined by consensus of repeated analyses.

Microscopic evaluations were periodically cross-checked among laboratory personnel to minimize observer bias. All laboratory equipment was maintained and calibrated according to routine institutional quality-assurance procedures.

### Data management and statistical analysis

All study data were recorded in Microsoft Excel spreadsheets immediately after collection to minimize transcription errors. Datasets were subsequently reviewed for completeness and consistency before being imported into IBM SPSS Statistics version 25.0 (IBM Corp., Armonk, NY, USA) for analysis.

Descriptive statistics were used to summarize the distribution of animals, farms, and samples included in the study. No missing-data imputation was required, and all analyses were performed using complete-case data.

Denominators for prevalence estimation were defined a priori as quarters for quarter-level mastitis prevalence, cows for animal-level prevalence, farms for herd-level prevalence, and fecal samples for parasitological prevalence. The clustering of quarters within cows and of cows within farms was acknowledged; however, cluster-adjusted confidence intervals (CIs) were not applied due to the study’s descriptive nature and limited sample size. Therefore, prevalence estimates were reported at the quarter, animal, and herd levels without adjustment for clustering; this limitation should be considered when interpreting them.

To improve statistical rigor, all prevalence estimates were accompanied by 95% CIs calculated using the Wilson score method, a non-Wald approach for binomial proportions.

Bacteriological culture results from mastitic milk were analyzed by calculating the frequency and proportion of bacterial isolates at the species level to identify the predominant pathogens associated with subclinical mastitis. Similarly, parasitological findings were summarized according to parasite species. No inferential or multivariable analyses were performed because the primary objective of this study was to generate descriptive epidemiological estimates rather than to identify or model risk factors. Study procedures and reporting were conducted in accordance with the Strengthening the Reporting of Observational Studies in Epidemiology–Veterinary (STROBE-Vet) recommendations for observational veterinary studies.

## RESULTS

### Animal and farm characteristics

The descriptive characteristics of animals and farms included in the study are presented in Table 1. Overall, the study population reflected typical smallholder dairy production systems characterized by traditional management practices and predominantly manual milking.

**Table 1:** Descriptive characteristics of animals and farms included in the study in Cendana District, Enrekang Regency, South Sulawesi. A. Animal level characteristics (n = 94).

No.	Variable	Category	Frequency (n)	Percentage (%)
1	Age (years)	Mean ± SD	4.9 ± 0.23	–
		Range	1–11	–
2	Parity	Median (range)	2 (0–6)	–
3	Lactation status	Lactating cows	73	77.7
		Non-lactating cows	21	22.3

A total of 94 dairy cattle were included in the study (Table 1A). The mean age of the animals was 4.9 ± 0.23 years, with a range of 1–11 years, indicating a population comprising both young and mature animals. The median parity was 2 (range: 0–6), reflecting variability in reproductive status from heifers to multiparous cows. Most animals were lactating (73/94; 77.7%), whereas 21 animals (22.3%) were non-lactating at the time of sampling. These characteristics indicate that the study population was predominantly composed of actively productive dairy cattle, which is relevant for interpreting mastitis- and productivity-related outcomes.

A total of 29 smallholder dairy farms were included in the study (Table 1B). All farms practiced semi-intensive management, with cattle housed in simple sheds located adjacent to households. Concrete flooring was used on all farms, and manure was removed manually daily. However, manure was frequently stored near housing facilities, contributing to poor drainage conditions and potentially increasing environmental contamination. Livestock water was sourced primarily from groundwater, with limited treatment before use. Milking was performed manually on all farms, and hygiene standards varied among producers. Collectively, these findings indicate that dairy production in the study area was characterized by small-scale, resource-limited management systems with variable hygiene and biosecurity practices.

**Table:** B. Farm-level characteristics (n = 29).

No.	Variable	Category	Frequency (n)	Percentage (%)
1	Housing type	Intensive	0	0.0
		Semi-intensive	29	100.0
2	Flooring materials	Concrete	29	100.0
		Soil/earthen	0	0.0
3	Water resources	River	2	6.9
		Groundwater	27	93.1
4	Manure management	Daily removal	29	100.0
		Irregular removal	0	0.0
5	Forage and feeding method	Grazing	0	0.0
		Cut-and-carry	29	100.0
6	Drainage condition	Good	12	41.4
		Poor	17	58.6
7	Milking method	Hand milking	29	100.0
		Machine-milking	0	0.0

### Serological detection of bTB

All 90 serum samples tested by ELISA for antibodies against *M. bovis* were negative. No serological evidence of exposure to *M. bovis* was detected among the sampled dairy cattle in Cendana Sub-district during the study period.

### Brucellosis screening

Serological screening of all 90 dairy cattle using RBT yielded negative results. No serum samples exhibited agglutination reactions indicative of antibodies against *B. abortus*. Consequently, no samples required confirmatory testing.

### Subclinical mastitis

**Prevalence of subclinical mastitis**: The CMT revealed a high prevalence of subclinical mastitis at quarter, animal, and herd levels (Table 2). At the quarter level, 112 of 289 quarters tested positive, corresponding to a prevalence of 38.75% (95% CI: 33.2%–44.6%). At the animal level, 55 of 73 lactating cows had at least one CMT-positive quarter, yielding a prevalence of 75.34% (95% CI: 64.2%–84.1%). At the herd level, 25 of 29 farms had at least one affected cow, corresponding to a prevalence of 86.21% (95% CI: 68.8%–95.0%).

**Table 2:** Prevalence of subclinical mastitis in smallholder dairy cattle based on CMT in Cendana District, Enrekang Regency.

Level of analysis	Total samples	Positive samples	Prevalence (%)	95% Confidence interval
Quarter	289	112	38.75	33.2–44.6
Animal	73	55	75.34	64.2–84.1
Herd	29	25	86.21	68.8–95.0

**Severity of subclinical mastitis:** The severity of subclinical mastitis, based on the CMT score distribution, is presented in Table 3. Most udder quarters were classified as negative, with 177 of 289 quarters (61.25%) receiving a score of 0. However, varying degrees of subclinical inflammation were observed. A total of 82 quarters (28.37%) were categorized as trace (+), indicating mild inflammatory changes, and 30 quarters (10.38%) as moderate (++). No quarters were assigned a severe (+++) score. These findings indicate that although advanced subclinical mastitis was not detected, a substantial proportion of quarters were affected by mild-to-moderate inflammation.

**Table 3:** Distribution of CMT scores reflecting subclinical mastitis severity in dairy cattle in Cendana District, Enrekang Regency.

CMT score	Number of quarters (n)	Percentage
0	177	61.25
+	82	28.37
++	30	10.38
+++	0	0
Total	289	100

**Bacteriological examination of mastitic milk:** Bacteriological culture followed by MALDI-TOF MS identification of milk samples from CMT-positive quarters (CMT score ≥2) yielded 28 bacterial isolates from 30 mastitic milk samples (Table 4). The most frequently identified pathogens were *Streptococcus uberis* (16.7%), *Streptococcus agalactiae* (13.3%), and *Staphylococcus aureus* (13.3%). Several other Gram-positive and Gram-negative bacterial species were detected at lower frequencies, whereas two samples yielded no bacterial growth.

**Table 4:** Bacterial species isolated from mastitic milk samples using MALDI-TOF MS in Cendana District, Enrekang Regency.

Bacterial species	Positive samples	Percentage
*Streptococcus agalactiae*	4	13.3
*Streptococcus infantarius*	1	3.3
*Klebsiella pneumoniae*	2	6.7
*Staphylococcus aureus*	4	13.3
*Staphylococcus chromogenes*	1	3.3
*Escherichia coli*	2	6.7
*Streptococcus uberis*	5	16.7
*Streptococcus equinus*	1	3.3
*Staphylococcus haemolyticus*	1	3.3
*Klebsiella oxytoca*	1	3.3
*Serratia marcescens*	1	3.3
*Lactococcus garviae*	2	6.7
*Pseudomonas aeruginosa*	1	3.3
*Lactococcus lactis*	1	3.3
*Macrococcus caseolyticus*	1	3.3
Negative culture	2	6.7
Total	30	100.0

Polymicrobial growth was identified in 6.7% (2/30) of culture-positive samples, indicating the presence of more than one bacterial species within the same quarter. Although most intramammary infections appeared to be associated with a single pathogen, the occurrence of polymicrobial infections suggests potentially complex pathogen interactions that may influence disease progression, diagnosis, and treatment outcomes.

### Gastrointestinal parasitism

**Figure 2:**
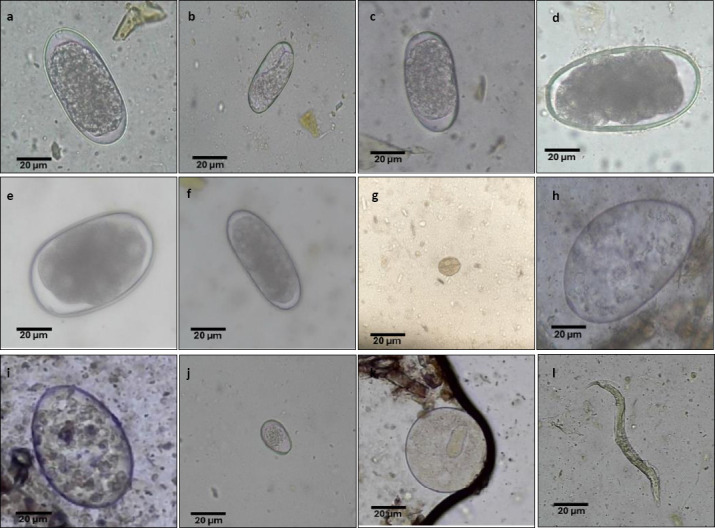
Representative photographs of gastrointestinal parasite eggs identified from fecal examination of dairy cattle in Cendana District, Enrekang Regency. (a) Strongyle-type egg, (b) *Strongyloides* sp., (c) *Ostertagia* sp., (d) *Nematodirus* sp., (e) *Oesophagostomum* sp., (f) *Cooperia* sp., (g) *Giardia* sp., (h) *Fasciola* sp., (i) *Paramphistomum* sp., (j) *Eimeria* sp., (k) *Balantidium* cyst, and (l) *Strongyloides* larva.

Fecal examination of 94 cattle demonstrated that 71 animals (75.53%) were positive for gastrointestinal endoparasites, including both helminths and protozoa (Table 5). As shown in Figure 2, the most frequently detected parasites were strongyle-type eggs (39.36%), followed by *Strongyloides* spp. (30.85%), *Eimeria* spp. (21.28%), and *Fasciola* spp. (14.89%). Several additional parasite taxa were identified at lower frequencies.

**Table 5:** Endoparasites identified from fecal examination of dairy cattle in Cendana District, Enrekang Regency (n = 94).

Parasite infection	Positive samples	Percentage
Gastrointestinal parasite eggs		
Strongyle	37	39.36
*Strongyloides* spp.	29	30.85
*Fasciola* spp.	14	14.89
*Paramphistomum* spp.	6	6.38
*Moniezia* spp.	5	5.32
*Ostertagia* spp.	4	4.26
*Nematodirus* spp.	3	3.19
*Ascaris* spp.	2	2.12
*Cooperia* spp., *Trichostrongylus* spp., *Oesophagostomum* spp., *Bunostomum* spp.	1	1.06
Protozoa		
*Eimeria* spp.	20	21.28
*Balantidium coli*	4	4.26
*Giardia* spp.	1	1.06

Multiple infections involving helminths and/or protozoa were common. Specifically, 32.98% (31/94) of examined samples contained more than one parasite type, indicating a substantial burden of co-infection. Such concurrent infections may impair nutrient utilization, reduce productivity, and increase susceptibility to other diseases through compromised immune function.

**Co-occurrence of infections**: Cross-tabulation analysis demonstrated substantial overlap between gastro-intestinal parasitism and subclinical mastitis. Among lactating cows, 56.16% (41/73) were concurrently affected by both conditions (Table 6). This finding indicates that co-infection represents a common health challenge within the study population. The observed pattern suggests that shared underlying factors, including inadequate nutrition, impaired immune function, and suboptimal herd management practices, may contribute to the simultaneous occurrence of these conditions in smallholder dairy systems.

**Table 6:** Co-occurrence of gastrointestinal parasitism and subclinical mastitis in dairy cattle in Cendana District, Enrekang Regency (n = 73).

Parasitism	Mastitis positive	Mastitis negative	Total
Positive	41	14	55
Negative	14	4	18
Total	55	18	73

## DISCUSSION

### Serological status of bTB and brucellosis

bTB appeared to be absent in the study area, as all 90 serum samples tested negative by ELISA for antibodies against *M. bovis*. This finding is encouraging because bTB remains an endemic zoonosis in many low- and middle-income countries, with bovine reservoirs posing risks to public health. Within Indonesia, reports suggest variable bTB prevalence in cattle, with a pooled prevalence of approximately 9.7%, although available data remain scarce and highly localized [9, 10]. The absence of seropositive cases in this study contrasts with recent reports indicating the presence of *M. tuberculosis* complex in dairy systems in South Sulawesi, including evidence of potential zoonotic transmission through milk [21]. However, those studies primarily used molecular or culture-based diagnostic approaches and often focused on high-risk populations or targeted surveillance settings. By contrast, the present study used ELISA as a serological screening tool in a cross-sectional survey of smallholder herds.

The complete absence of bTB seropositivity in Enrekang may reflect effective regional control, low cattle-to-cattle transmission, or limitations of serological sensitivity, particularly during early infection. However, the snapshot nature of cross-sectional serology must be acknowledged, and periodic surveillance using complementary diagnostic methods, including tuberculin skin testing, bacteriological culture, and molecular approaches, would strengthen confidence in these findings.

The absence of seropositive animals for brucellosis, as determined by RBT, suggests that *B. abortus* infection was not circulating among smallholder dairy cattle in Enrekang Regency at the time of sampling. This finding is important given the zoonotic nature of brucellosis and its well-documented effects on animal productivity and human health. However, these results contrast with reports from other regions of Indonesia, where brucellosis has been detected at varying prevalence levels. A meta-analysis of livestock brucellosis studies across Indonesia estimated a pooled prevalence of approximately 4.2%, with higher rates in provinces such as Central Java and West Java [8]. Similarly, retrospective analyses of national surveillance data from 2006 to 2020 documented sporadic outbreaks of *B. abortus*, underscoring its continued relevance in Indonesian livestock populations [9].

The absence of bTB and brucellosis in this study may reflect regional differences in livestock movement, biosecurity practices, and herd structure. However, these results should be interpreted with caution because serological screening methods may have limited sensitivity in detecting early or latent infections. Therefore, the absence of detected cases does not necessarily confirm the absence of disease and may also reflect under-detection. Further studies should incorporate more sensitive and complementary diagnostic approaches to better characterize the epidemiological status of these zoonotic diseases in the region.

### Burden of subclinical mastitis

In contrast to the absence of bTB and brucellosis, the high prevalence of subclinical mastitis observed in this study, namely 38.75% at the quarter level, 75.34% at the cow level, and 86.21% at the herd level, indicates a substantial udder health burden among smallholder dairy farms in Enrekang Regency. These findings are consistent with observations from East Java, where prevalence was reported at 66.72% at the quarter level and 68.18% at the cow level, indicating systemic udder health challenges across provincial settings in Indonesia [22]. Similarly, in Bogor, West Java, smallholder farms showed a cow-level prevalence of subclinical mastitis of 82.5%, further supporting the widespread nature of this problem in traditional dairy production systems [23]. In contrast, some regions, such as Deyeng Village in East Java, reported considerably lower prevalence (21.3%), potentially reflecting better management practices, including improved milking hygiene and dry-period treatments [24]. Although most published data originate from Java and other western regions of Indonesia, the present findings suggest that similar mastitis-related challenges exist in eastern regions such as South Sulawesi. The limited availability of region-specific epidemiological data from Sulawesi has previously restricted understanding of mastitis dynamics in these systems. Therefore, this study provides important baseline evidence for the region.

### Bacterial pathogens associated with mastitis

The bacterial species isolated in this study showed that *S. uberis* (16.7%), *S. agalactiae* (13.3%), and *S. aureus* (13.3%) were predominant, indicating the involvement of both environmental and contagious transmission routes. This finding is consistent with reports from other regions. For example, a Bangladeshi study identified *S. uberis* and *S. agalactiae* among the leading pathogens in clinical mastitis, with substantial virulence gene profiles [25]. Although that study focused on clinical mastitis, it demonstrates the broader relevance of *Streptococcus* species in mastitis epidemiology.

Studies conducted in Tanzania have also reported significant proportions of *S. aureus*, *Streptococcus* spp., and *Escherichia coli* in mastitic milk, reinforcing the common occurrence of these pathogens across diverse smallholder systems [26]. The isolation of *Klebsiella pneumoniae*, *E. coli*, and *Pseudomonas aeruginosa* in the present study, although at lower frequencies, is consistent with global patterns of environmental coliform mastitis agents, which are often associated with poor bedding and barn sanitation rather than milking hygiene alone [27–29].

Two samples showed no bacterial growth (6.7%), suggesting possible non-infectious causes or limitations of culture sensitivity. This finding mirrors similar observations from Tanzania, where a subset of CMT-positive cases did not yield detectable pathogens [26]. These results highlight the complexity of interpreting subclinical mastitis status and emphasize the potential value of somatic cell count methods or molecular detection techniques in future surveillance. In this context, negative culture results may also reflect the translocation of bacterial components, rather than viable organisms, from distant anatomical sites such as the uterus, as demonstrated in previous studies [30–32].

### Gastrointestinal parasitism in smallholder dairy cattle

The high prevalence of gastrointestinal parasitism observed in this study (75.53%), with dominant infections caused by strongyle-type eggs (39.36%), *Strongyloides* spp. (30.85%), *Eimeria* spp. (21.28%), and *Fasciola* spp. (14.89%), underscores the substantial parasitic burden affecting smallholder dairy cattle in Enrekang Regency. These results are consistent with recent meta-analyses and field studies in Indonesia. A systematic review reported pooled prevalence rates of gastrointestinal parasites in cattle exceeding 70%, with strongyles and *Eimeria* spp. among the most frequently detected genera [33]. In East Java, surveys of Madura cattle showed diverse parasite communities, including *Fasciola* spp. and *Paramphistomum* spp., reflecting the broad spectrum of helminths and protozoa affecting Indonesian herds [34]. Compared with the nationwide survey [35], which reported an overall prevalence of 24.2% for gastrointestinal nematodes across 15 provinces, the burden observed in Enrekang appears substantially higher. That survey documented strongyle infections in 19.1% of cattle, with provincial peaks reaching 52.8% in West Nusa Tenggara and 40% in Southeast Sulawesi. The 39.36% strongyle prevalence observed in South Sulawesi is close to these provincial hotspots, suggesting that the humid climate and traditional smallholder practices in Sulawesi provide favorable conditions for parasite persistence.

*Fasciola* spp. were detected in 14.89% of fecal samples. This prevalence is epidemiologically important because fasciolosis is widely recognized as one of the most economically significant trematode infections in tropical livestock systems. Interestingly, the prevalence observed in Enrekang was lower than figures reported in certain endemic zones of Central Java, Ambon, and West Sumatra, where rates can exceed 20% [36–38]. This difference may reflect regional variation in snail habitats, irrigation intensity, and grazing systems. However, detecting fasciolosis in nearly one in seven animals remains important, particularly in smallholder settings where even modest productivity losses can cause substantial economic hardship.

This study focused exclusively on smallholder dairy cattle in Enrekang Regency, where animals are typically maintained under tie-stall or simple housing systems and milked by hand. The cattle were managed intensively, confined to housing areas, and fed cut-and-carry forage rather than being allowed to graze freely. Therefore, exposure to parasites was likely shaped primarily by housing hygiene, bedding moisture, and water management within pens rather than by pasture contamination.

### Management, social, and economic implications

From a social and cultural perspective, the high prevalence of both subclinical mastitis and gastrointestinal parasitic infections can be attributed to traditional management practices such as hand milking, limited hygiene practices, simple housing systems, poor drainage, and inadequate manure management [22–24, 26, 35, 39–41]. The combination of a high mastitis burden, driven by both environmental and contagious pathogens, and widespread parasitism under cut-and-carry feeding systems demonstrates how local management practices shape disease dynamics in eastern Indonesia. These patterns differ from better-documented systems in Java and underscore the need for region-specific interventions. These conditions are often under-recognized by farmers due to subtle clinical signs, leading to limited preventive action.

Economically, the impact is considerable because mastitis reduces milk yield and quality, whereas parasitic infections impair feed efficiency, growth, and fertility. In addition, fasciolosis may cause liver condemnation and direct financial losses at slaughter [36–38, 42–44]. For smallholder families relying on daily milk sales for income, even modest reductions in productivity can be significant. These findings underscore the need for integrated herd health interventions combining improved milking hygiene, housing sanitation, strategic deworming, and farmer education to protect both livelihoods and food security.

### Practical recommendations

Based on the findings, targeted interventions are needed to address the identified health constraints. For mastitis control, improving milking hygiene practices, including proper udder cleaning and post-milking teat disinfection, is essential to reduce transmission of environmental and contagious pathogens such as streptococci and staphylococci. Routine screening using CMT should be encouraged to facilitate early detection and management of subclinical cases.

For parasitic infections, strategic deworming programs should be combined with improved environmental management, including better drainage and sanitation, to reduce exposure to infective stages. In areas where suitable ecological conditions exist, control of intermediate hosts should also be considered for parasites such as *Fasciola* spp. These measures are essential to improve cattle health and productivity in smallholder dairy systems.

### Strengths and limitations

This study provides one of the first integrated multi-pathogen epidemiological surveys of smallholder dairy cattle in Enrekang Regency, South Sulawesi. Its strengths include the simultaneous assessment of zoonotic bacterial diseases, subclinical mastitis, and gastrointestinal parasitism using serological, microbiological, and parasitological approaches. The use of MALDI-TOF MS for bacterial identification also strengthened pathogen-level interpretation of mastitis cases and provided more precise bacterial identification than conventional phenotypic approaches.

Nevertheless, this study has several limitations. First, the use of purposive sampling may have introduced selection bias and may limit the generalizability of the findings to the broader dairy cattle population. Second, the cross-sectional design provides only a snapshot of herd health status and cannot capture seasonal variation or longitudinal trends. Third, parasitological assessment was limited to qualitative detection because quantitative fecal egg counts were not performed. Fourth, reliance on serological methods for bTB and brucellosis may have underestimated true prevalence, particularly in early or latent infections. Finally, although mastitis and parasite prevalence were quantified, risk-factor analyses were not performed, limiting the ability to identify specific management practices associated with disease occurrence. Future studies should adopt longitudinal designs, include seasonal sampling, incorporate molecular and culture-based confirmation for zoonotic diseases, and apply multivariable analyses to better understand causal pathways and management-related risk factors.

## CONCLUSION

This study represents the first integrated multi-pathogen epidemiological survey of smallholder dairy cattle in Enrekang Regency, South Sulawesi, simultaneously assessing zoonotic bacterial diseases, subclinical mastitis, and gastrointestinal parasitism. The findings demonstrated the absence of serological evidence of bTB and brucellosis among the sampled cattle population. In contrast, subclinical mastitis was highly prevalent, affecting 38.75% of udder quarters, 75.34% of lactating cows, and 86.21% of herds. The predominant mastitis-associated pathogens were *S. uberis*, *S. agalactiae*, and *S. aureus*, indicating the involvement of both environmental and contagious transmission pathways. Gastrointestinal parasitism was also widespread, with an overall prevalence of 75.53%, dominated by strongyle-type eggs, *Strongyloides* spp., *Eimeria* spp., and *Fasciola* spp. Furthermore, more than half of the lactating cows exhibited concurrent gastrointestinal parasitism and subclinical mastitis, highlighting the cumulative health burden affecting smallholder dairy systems.

The results indicate that endemic production-limiting diseases currently represent a greater challenge to dairy productivity in the study area than the investigated zoonotic bacterial diseases. From a practical perspective, these findings emphasize the importance of strengthening routine herd health management through improved milking hygiene, enhanced housing sanitation, effective manure management, regular mastitis screening, strategic parasite control programs, and farmer education. Such measures are essential for improving milk quality, animal welfare, and farm profitability while supporting sustainable dairy production in eastern Indonesia.

Future investigations should incorporate longitudinal study designs, seasonal monitoring, quantitative parasitological assessments, molecular confirmation of zoonotic infections, and multivariable analyses to identify management-related risk factors. Such studies would provide a more comprehensive understanding of disease transmission dynamics and support the development of targeted intervention strategies.

Overall, the present findings provide important baseline epidemiological evidence for an emerging dairy production region in Indonesia and highlight the urgent need for integrated herd health programs focused on mastitis prevention and parasite control. Addressing these endemic health constraints will be critical for improving dairy cattle productivity, enhancing farmer livelihoods, and supporting the long-term sustainability of smallholder dairy systems in South Sulawesi.

## DATA AVAILABILITY

All data supporting the results are available in the manuscript.

## AUTHOR’S CONTRIBUTION

FYP, AAPJW, and MAN: Conceptualization and study design. FYP, AAPJW, MAN, MMN, AMK, ZM, and SY: Investigation, data collection, and data analysis. FYP, EVGK, and EJE: Supervision. FYP, AAPJW, MAN, EVGK, and EJE: Manuscript writing, review, and revision. All authors have read and approved the final version of the manuscript.
